# Coevolution in RNA Molecules Driven by Selective Constraints: Evidence from 5S rRNA

**DOI:** 10.1371/journal.pone.0044376

**Published:** 2012-09-04

**Authors:** Nan Cheng, Yuanhui Mao, Youyi Shi, Shiheng Tao

**Affiliations:** 1 StateKey Laboratory of Crop Stress Biology in Arid Areas and College of Life Sciences, Northwest A&F University, Yangling, People’s Republic of China; 2 Bioinformatics Center, Northwest A&F University, Yangling, People’s Republic of China; 3 College of Science, Northwest A&F University, Yangling, People’s Republic of China; Tel Aviv University, Israel

## Abstract

Understanding intra-molecular coevolution helps to elucidate various structural and functional constraints acting on molecules and might have practical applications in predicting molecular structure and interactions. In this study, we used 5S rRNA as a template to investigate how selective constraints have shaped the RNA evolution. We have observed the nonrandom occurrence of paired differences along the phylogenetic trees, the high rate of compensatory evolution, and the high TIR scores (the ratio of the numbers of terminal to intermediate states), all of which indicate that significant positive selection has driven the evolution of 5S rRNA. We found three mechanisms of compensatory evolution: Watson-Crick interaction (the primary one), complex interactions between multiple sites within a stem, and interplay of stems and loops. Coevolutionary interactions between sites were observed to be highly dependent on the structural and functional environment in which they occurred. Coevolution occurred mostly in those sites closest to loops or bulges within structurally or functionally important helices, which may be under weaker selective constraints than other stem positions. Breaking these pairs would directly increase the size of the adjoining loop or bulge, causing a partial or total structural rearrangement. In conclusion, our results indicate that sequence coevolution is a direct result of maintaining optimal structural and functional integrity.

## Introduction

Selective constraints often operate on an entire molecular system, and require coordinated changes of its components. Such long-term interactions obviously occur between molecules within a cell, and between residues within a molecule. Examples of such interactions include the coordinated changes of amino acid residues in a protein molecule [1], [2], [3], [4], [5], compensatory substitution in RNA molecules [6], [7], [8], [9], intramolecular interactions [10], [11], [12], compensatory *trans* and *cis* mutations within a transcriptional network [13], and the copresence of enzymes in the same metabolic pathway [14], [15].

The secondary structures of rRNAs are remarkably uniform across taxa. This level of conservation is achieved by a special pattern of base changes known as compensatory mutations [16]. RNA molecules exhibit strong signs of coevolution, especially between Watson-Crick pairs of nucleotides within stems. The deleterious effect of base substitution at a given site can be suppressed by a compensatory second-site substitution [17], [18], [19]. Therefore, revealing intra-molecular coevolution is important for understanding of various structural and functional constraints acting on RNA molecules, which also has potential use in predicting molecular interactions and structures [8].

To date, various methods have been used to identify coevolution of genes. Some studies have measured coevolution by the similarity in absolute evolutionary rate (ER) or dN/dS (the rate of nonsynonymous substitution rate divided by the rate of synonymous substitution) [20], [21], [22], correlative ER or dN/dS [23]. Others have applied correlation metrics to detect the covariation of sequences, such as correlation coefficients [24], mutual interdependency [25], and mutual information (MI) [26], [27], [28]. Besides, some model-based methods rely on standard Markov models of sequence evolution, and take substitution probabilities among states or the among-site rate variation into account [29], [30], [31], [32], [33].

These studies focused on second-site substitutions that directly restore the disrupted Watson-Crick interaction (e.g. GC↔GU↔AU). Most of these approaches have assumed that mutations disrupting the base-pairing of a functionally important RNA stem are deleterious, while the deleterious effect may be overcome by a second compensatory mutation in the other half of the stem, which restores the potential for base-pairing [34]. On a larger evolutionary scale, however, such a mechanism failed to explain all observed patterns of coevolution. Moreover, the intricate relations between sequence coevolution and various selective constraints are worth pursuing at a deeper level.

Here, we focus on 5S rRNAs, a class of non-protein coding RNAs with well-studied structure and function, to investigate how selective constraints shape RNA evolution. We infer the substitution histories of 5S rRNA sequences and investigate how selective constraints might have influenced the rate and pattern of evolution in different structural regions of 5S rRNA.

## Materials and Methods

### Sequences and Structure

**Figure 1:**
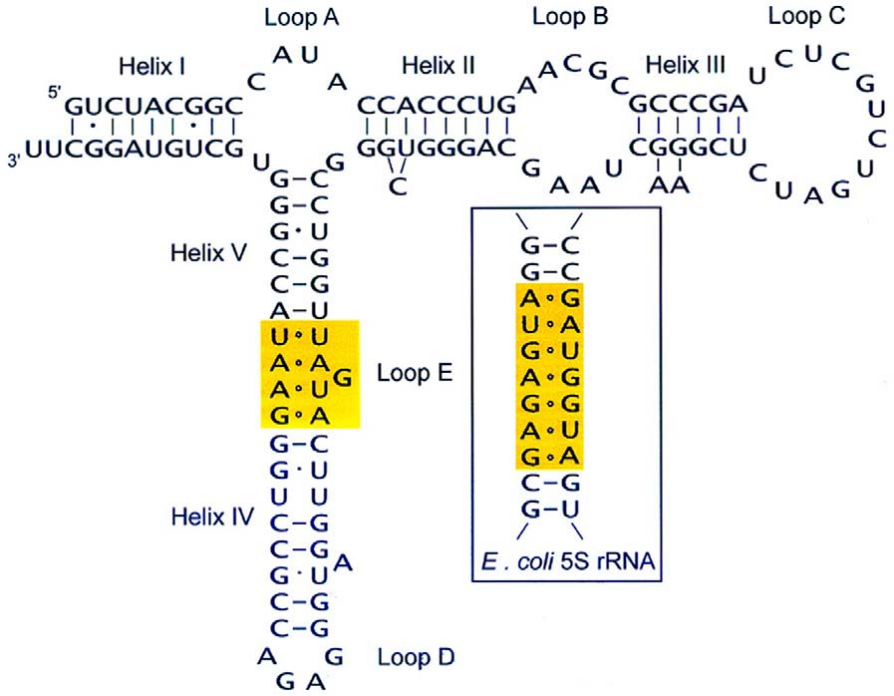
Secondary structure of human 5S rRNA. In all organisms, the structure consists of five double-stranded regions (I–V) and five loops (A–E). Loop E, which differs between eukaryotic and eubacterial 5S rRNAs, is highlighted in yellow.

Aligned small subunit (SSU) and large subunit (LSU) rRNA sequences were obtained from the SILVA database [35]. Alignments were inspected by eye and slightly modified. All ambiguous aligned sites were discarded from the analysis. The two data sets were then concatenated to estimate a common phylogeny, in an attempt to enhance the ratio of signal to noise and thus more reliably recover the “true” organismal phylogeny. The aligned 5S sequences and consensus secondary structure information were downloaded from the 5S Ribosomal RNA database [36]. 5S rRNA has a length of ∼120 nt and a highly conserved structure, which consists of five stems (helices I-V), two hairpin loops (C and D), two internal loops (B and E) and a hinge region (loop A) forming the three helix junction (Figure 1). A total of 153 species including 39 bacteria, 31 animals, 37 plants, and 46 fungi were used for our analysis.

### Phylogenetic Reconstruction

Nucleotide substitution models and parameters were estimated using JMODELTEST [37] using default settings. Phylogenetic tree reconstruction was then constructed by employing the maximum likelihood (ML) method implemented in PhyML 3.0 [38] and Bayesian approaches in MrBayes3.1 [39]. For PhyML analysis, the robustness of the statistical support for the tree branch was evaluated by 100 bootstrap replicates. For MrBayes analysis, 2,000,000 generations were used for 4 simultaneous Markov chains. Trees were sampled every 100 generations, and the last 10,000 trees (well after the chain reached stationarity) were used for inferring Bayesian posterior probability.

### Ancestral States Reconstruction

Ancestral 5S rRNA sequences of all interior nodes in the phylogenetic trees were statistically inferred from the present-day sequences by using the Empirical Bayesian (EB) method under the best fitting model. The EB analysis was implemented by the PAML program [40]. Marginal posterior probabilities at each site were also calculated in this program. The accuracy of the ancestral state reconstruction might depend on the underlying model used in the reconstruction. Therefore, besides the GTR model, we also performed the analysis using 7 other models implemented in the PAML package to confirm the robustness of our results with regard to the accuracy of the ancestral state reconstruction.

### Estimation of Substitution Rates

We used PAML to estimate the site-specific evolutionary rates, which we report as an indication of selection of constraint. This analysis was implemented by the baseml program of PAML, using the best nucleotide substitution model suggested by JMODELTEST. The site-specific rates inferred here are not absolute evolutionary rates that require knowledge of divergence times, but rather they represent a comparative quantity.

**Figure 2:**
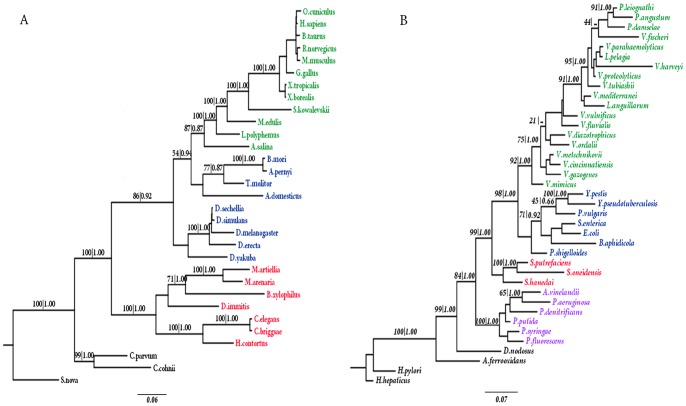
The phylogenetic tree constructed by the concatenated sequences of SSU and LSU rRNA based on GTR+G model. Numbers indicate the bootstrap scores for ML (left) and Bayesian posterior probabilities for Bayesian (right) that supported the indicated node. Taxon names are color coded according to the taxonomic order designation at NCBI. (A)Animal. Green: chordata, blue: arthropoda, red: nematoda, black: outgroup. (B) γ-proteobacteria. Green: virionales, blue: enterobacteriales, red: alteromonadales, violet: pseudomonadales, black: outgroup.

### Measuring Coevolution

Firstly, we used the clustering approach implemented in the CoMap program [41] to detect the coevolution within our 5S rRNA sequences. This approach searches for ancestral co-substitution or for compensatory changes by correlating nucleotide substitution. Coevolution was detected as non-independent evolution among sites. The degree of correlated evolution was estimated based on the correlation coefficient of the substitution vectors. To assess the significance of inferred clusters, a parametric bootstrap with 10,000 replicates was used to generate the joint null distribution of minimum site variability together with coevolution or compensation statistic 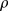, as described by Dutheil and Galtier [41]. Clusters with 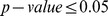 were considered to be evolving non-independently. Secondly, we implemented a corrected mutual information method (MIp) [28] for the coevolution detection. MIp methods use the phylogenetic signal available to assess the significance of coevolution, but do not assume a particular phylogenetic tree. The MIp computations were implemented in C++ as a dedicated program named MICA (mutual information coevolution analysis) [42].

**Figure 3:**
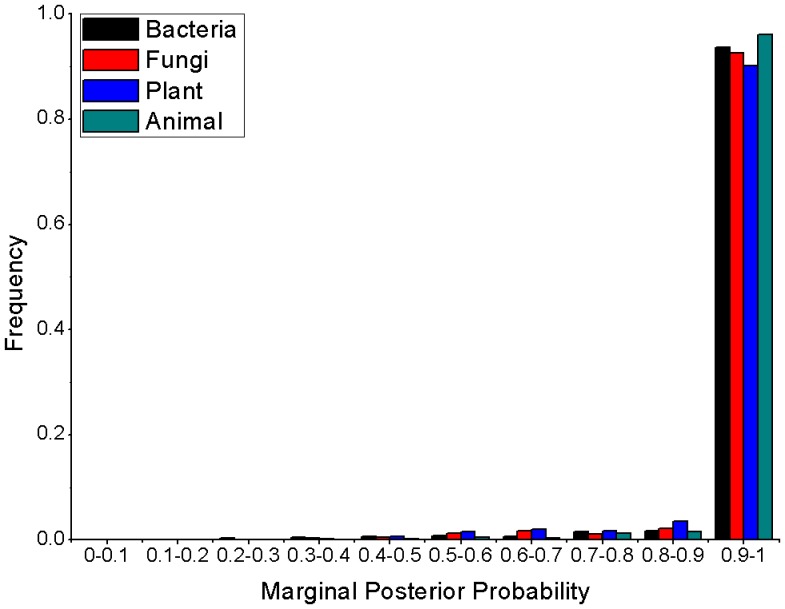
The accuracy of ancestral states reconstruction. Frequencies of marginal posterior probabilities calculated for the most likely nucleotide reconstruction at each site of the ancestral 5S rRNA sequence under the GTR+G model.

**Table 1:** Coevolving positions detected in eukaryotic and eubacterial 5S rRNA.

Species	Helix	Alignment	Positions	CoMap	MIp
	Helix I	7–134	Near a bulge	4.78×10^−4^	1.08×10^−2^
		9–133	Near a bulge	5.05×10^−3^	1.80×10^−3^
		10–132	Other states	1.95×10^−3^	8.41×10^−3^
		11–131	Near loop A	1.85×10^−4^	6.47×10^−4^
		12–130	Near loop A	9.80×10^−3^	1.80×10^−3^
	Helix II	18–75	Near loop A	1.02×10^−3^	1.80×10^−3^
		20–72	Near a bulge	5.05×10^−3^	3.60×10^−3^
		28–67	Near loop B	1.60×10^−2^	1.77×10^−2^
	Helix III	35–62	Near loop B	4.78×10^−4^	1.62×10^−3^
Eukaryotes		36–61	Near loop B	8.51×10^−5^	3.78×10^−3^
		37–57	Near a bulge	2.55×10^−4^	5.04×10^−3^
		38–56	Near a bulge	9.80×10^−3^	3.60×10^−3^
		39–55	Near loop C	1.02×10^−3^	6.25×10^−3^
		40–54	Near loop C	4.78×10^−4^	1.65×10^−3^
	Helix IV	90–111	Near loop D	4.78×10^−4^	1.62×10^−3^
		92–109	Other states	5.05×10^−3^	1.08×10^−2^
		93–108	Other states	5.05×10^−3^	4.31×10^−3^
		94–107	Near a bulge	2.32×10^−4^	1.26×10^−3^
		97–104	Near loop E	8.51×10^−5^	4.20×10^−3^
		98–103	Near loop E	8.51×10^−5^	1.48×10^−3^
	Helix V	77–127	Near loop A	4.61×10^−4^	1.26×10^−3^
		80–118	Near a bulge	1.02×10^−3^	1.65×10^−3^
		82–117	Near a bulge	4.63×10^−4^	6.49×10^−3^
	Helix I	8–153	near a bulge	5.38×10^−4^	4.42×10^−3^
		14–145	near a bulge	3.03×10^−3^	6.21×10^−4^
		15–144	near a bulge	5.37×10^−3^	7.87×10^−3^
		16–143	other states	5.00×10^−3^	2.47×10^−3^
		17–142	near loop A	1.12×10^−2^	1.85×10^−2^
		18–141	near loop A	8.51×10^−4^	2.13×10^−2^
	Helix II	27–86	near loop A	4.78×10^−3^	8.33×10^−3^
		31–81	near a bulge	2.62×10^−3^	3.23×10^−3^
Eubacteria		35–79	near a bulge	1.93×10^−3^	1.24×10^−3^
	Helix III	48–66	near loop C	1.77×10^−3^	6.21×10^−4^
		49–65	near loop C	2.62×10^−3^	6.21×10^−4^
	Helix IV	101–124	near loop E	7.06×10^−3^	6.21×10^−4^
		105–121	near a bulge	9.64×10^−5^	1.24×10^−3^
		106–119	other states	1.47×10^−3^	1.24×10^−3^
		107–118	other states	1.54×10^−3^	5.83×10^−3^
		108–117	near loop D	3.67×10^−4^	1.24×10^−3^
		109–116	near loop D	2.75×10^−4^	6.21×10^−4^
	Helix V	90–134	near loop E	6.75×10^−4^	2.92×10^−3^

### Evolution of Compensatory Mutations

The evolution of RNA molecules can proceed through a characteristic substitution pattern that maintains the pairing capability between paired bases. Four kinds of substitution patterns are possible. The most common is the switch between an AU and GC pair (AU↔GC), through the intermediate state of AC or GU. The three other types of switch are AU↔UA, GC↔CG and AU↔CG, with the intermediate states being AA or UU, GG or CC, and AG or UC, respectively.

Using the inferred phylogenetic trees, we first attempted to show the nonrandom occurrence of sequence differences that maintain base pairing. Through examination of the changes that occurred between nodes on these trees, we could observe the behavior of paired sequence differences. For all cases in which both of the paired sites change, if the changes are neutral, only one-third of the second changes would restore base pairing. The difference between expected and observed distributions was analyzed statistically using the χ^2^-test.

We then investigated the evolutionary interdependence of two substitutions involved in a Watson-Crick switch. We recorded a Watson-Crick switch when two lineages harbored different Watson-Crick pairs of nucleotides at a pair of interacting sites and the switch between them was caused by exactly two substitutions, as judged from the reconstructed ancestral states. If two substitutions are selectively neutral, they are expected to occur independently, without clustering on the phylogenetic tree. The extent of clustering can be characterized by the ratio of the numbers of terminal to intermediate states (the terminal-to-intermediate ratio, TIR) in the last common ancestor (LCA) of the species [43].The LCA state separated by a Watson-Crick substitution can either be identical to the terminal state (AU or GC), or coincide with the intermediate state (e.g. AC or GU). If all substitutions are selectively neutral, the TIR is expected to be 1∶1. A TIR with more frequent LCA terminal states may indicate clustering of the two substitutions involved in a Watson-Crick substitution and positive selection is involved in the evolution of interacting pairs.

**Figure 4:**
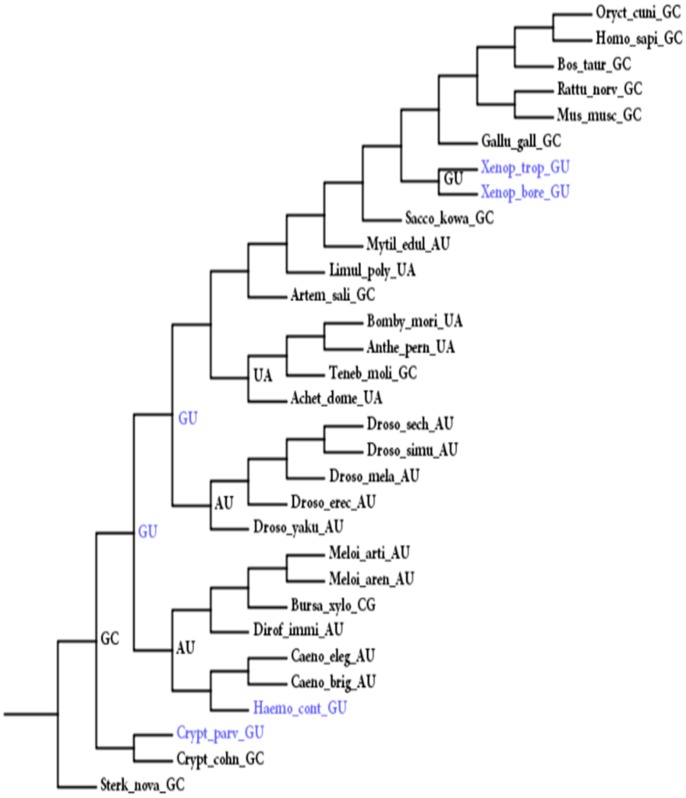
An example of coevolving pairs 8-111 detected in animal 5S rRNA sequences. The disruption of GC pair was compensated by a G8A substitution that created an AU pair or by a U111C substitution that restored the GC pair. Intermediate states are shown in blue.

**Table 2:** Number of compensatory switches in 5S rRNAs.

Species	compensatory switches	Ancestral state(Terminal)		Ancestral state(intermediate)		Multiple changes
Eubacteria	AU↔GC	AU	GC	GU	AC	
		6	32	13	0	2
	AU↔CG	AU	CG	AG	UC	
		5	0	0	2	0
	AU↔UA	AU	UA	UU	AA	
		0	0	0	0	1
	GC↔CG	CG	GC	CC	GG	
		0	0	0	0	1
Fungi	AU↔GC	AU	GC	GU	AC	
		27	33	5	5	1
	AU↔CG	AU	CG	AG	UC	
		4	6	0	0	2
	AU↔UA	AU	UA	UU	AA	
		8	4	0	0	2
	GC↔CG	CG	GC	CC	GG	
		12	9	1	4	0
Plants	AU↔GC	AU	GC	GU	AC	
		7	31	5	0	0
	AU↔CG	AU	CG	AG	UC	
		2	0	0	0	1
	AU↔UA	AU	UA	UU	AA	
		0	0	0	0	0
	GC↔CG	CG	GC	CC	GG	
		4	10	0	1	0
Animals	AU↔GC	AU	GC	GU	AC	
		6	21	8	0	2
	AU↔CG	AU	CG	AG	UC	
		4	3	0	0	0
	AU↔UA	AU	UA	UU	AA	
		7	1	0	0	0
	GC↔CG	CG	GC	CC	GG	
		6	7	0	0	0

### Simulation of 5S rRNA Sequence Evolution along the Phylogenetic Trees

We tested the methods in this paper on randomly generated sequence data. Simulated data sets of nucleotide sequences were generated along the ML trees 1000 times (the null hypothesis). We simulated the data using the ML parameters of the substitution model inferred from the real sequences using the Seq-Gen program [44]. We then extended our methods on the resulting simulated data sets. For each of these simulated sequences, we also predicted the structure using the program RNAfold [45], [46]. Seq-Gen, although widely used, does not take into consideration the base pairing in the RNA structure. To this end, we used PHASE2.0 [47], which allows the labeling of RNA secondary structure into classes. RNA7D model was used for the simulation of stem regions. RNA7D model, which groups all noncanonical bases into a single mismatch class, offers a reasonable trade-off between the generality and numbers of parameters suitable for the size of data sets used in this study. 1000 repetitive simulations were performed by the PHASE program following the phylogenetic tree inferred from the real sequences. These simulated sequences were also used to rule out the possibility of an artifact due to model misspecification.

**Figure 5:**
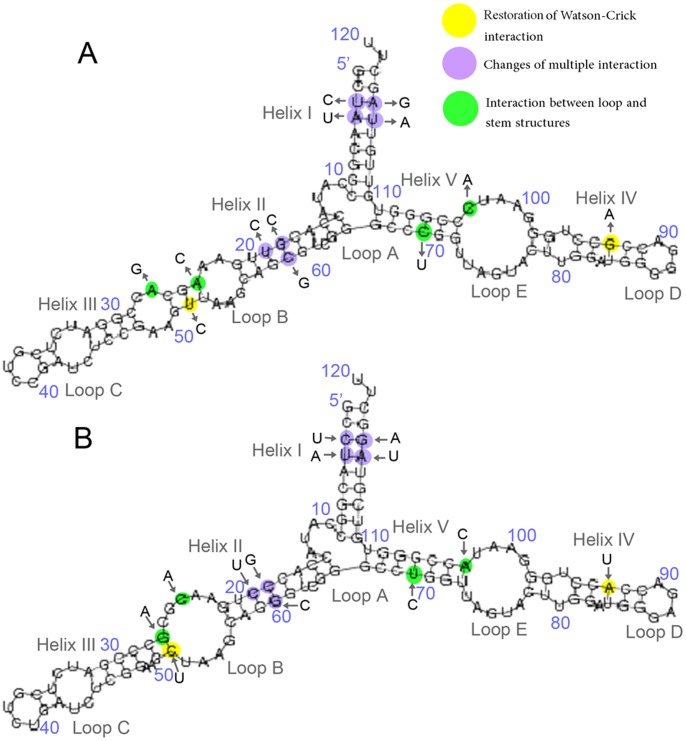
Evolutionary secondary structure maps of 5S rRNA. (A) Cenancestor 5S rRNA structure. (B) Human 5S rRNA secondary structure. Compensatory substitutions that restored Watson-Crick pairs were shown in yellow. Coevolutionary interactions of multiple stem pairs were shown in violet and interactions of stem and loop structures were shown in green.

### RNA Secondary Structure Prediction

The secondary structures of *Escherichia coli* [48] and *Homo sapiens* [49] 5S rRNA sequences were used as references for the determination of prokaryotic and eukaryotic structural pairs, respectively. To trace the evolution of 5S rRNA structure, we predicted the ancestral structures of all interior nodes in the phylogenetic trees using RNAfold (Vienna RNA package 1.8.5) [45], [46], with the consensus secondary structures as the constraints.

**Table 3:** Observed and Expected Substitutions in 5S rRNAs.

Data site and substitutions		Differences		χ^2^-test
		Paired	Unpaired	
Eubacteria	Observed	20	5	6.29×10^−4^
	Expected	8	17	
Fungi	Observed	38	15	9.97×10^−5^
	Expected	18	35	
Plants	Observed	15	1	2.61×10^−4^
	Expected	5	11	
Animals	Observed	19	8	6.46×10^−3^
	Expected	9	18	

## Results

### Phylogenetic Reconstruction

The GTR (general time reversible)+G (gamma distribution) evolutionary model [50] was selected as the optimal nucleotide substitution model. Phylogenetic tree reconstruction was performed using both ML and Bayesian methods with the GTR+G model. Both the ML and the Bayesian analyses converged on nearly identical topologies with proportionately similar support levels (Figure 2, Figure S1). Our results are consistent with those of some previous phylogenetic studies [51], [52].

### The Accuracy of Ancestral State Reconstruction

The EB method produced accurate reconstructions, with an average accuracy rate of 97.5±2.41% (Mean±SEM of the accuracies of the ensemble of reconstructions) of all nodes at all sites correctly reconstructed. Across all reconstructed sites, marginal posterior probabilities tended to be above 0.9 for the EB analysis under the GTR+G model (Figure 3). For all tested models used by ML analysis, reconstructions of the ancestral 5S rRNAs were found to be in agreement at >98% of nucleotide sites, suggesting the robustness of ancestral inference (Table S1).

**Figure 6:**
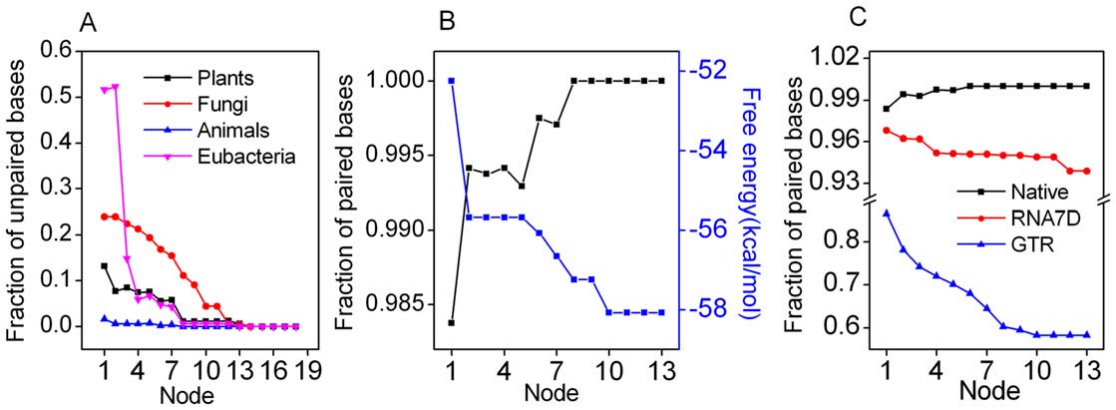
Compensatory substitutions that maintain base pairing contribute significantly to the stabilization of RNA structure. (A) Changes in unpaired bases within stems during the evolution of 5S rRNA. (B) Evolutionary secondary structure from ancestor to human 5S rRNA. (C) Changes in paired bases within stems of native and simulated 5S rRNA sequences.

**Table 4:** Stability of animal 5S rRNA secondary structures.

Species	Real ΔG	Mean ΔG	p-value^a^	Species	Real ΔG	Mean ΔG	p-value
*A.domesticus*	−53.90	−39.8±5.52	0.004	*G.gallus*	−53.90	−38.8±5.48	0.003
*A.pernyi*	−53.90	−39.6±5.59	0.006	*H.sapiens*	−47.00	−38.5±5.66	0.001
*A.salina*	−53.50	−40.6±5.61	0.005	*H.contortus*	−52.32	−38.7±5.56	0.010
*B.xylophilus*	−41.90	−37.6±5.61	0.022	*L.polyphemus*	−52.32	−40.5±5.44	0.002
*B.mori*	−59.60	−39.7±5.66	0.011	*M.artiellia*	−43.20	−38.1±5.82	0.019
*B.taurus*	−48.12	−38.5±5.65	0.001	*M.musculus*	−53.90	−38.5±5.69	0.001
*C.elegans*	−38.50	−38.0±5.63	0.046	*M.edulis*	−50.16	−40.2±5.62	0.005
*C.briggsae*	−41.80	−38.0±5.61	0.024	*M.arenaria*	−44.20	−38.0±5.55	0.014
*C.cohnii*	−45.80	−43.9±5.17	0.000	*O.cuniculus*	−53.90	−38.5±5.66	0.001
*D.sechellia*	−40.26	−37.9±5.67	0.001	*R.norvegicus*	−53.90	−38.5±5.73	0.001
*D.yakuba*	−57.40	−37.7±5.57	0.001	*S.nova*	−44.80	−46.8±5.00	0.065
*D.erecta*	−56.20	−37.2±5.76	0.001	*S.kowalevskii*	−52.50	−38.9±5.62	0.000
*D.melanogaster*	−52.32	−37.3±5.71	0.004	*T.molitor*	−55.22	−40.7±5.75	0.001
*D.immitis*	−49.80	−38.8±5.69	0.008	*X.tropicalis*	−52.32	−39.1±5.60	0.001
*D.simulans*	−48.90	−37.9±5.61	0.001	*X.borealis*	−52.60	−39.0±5.58	0.001

Means±SEM are shown.

a The proportion of native 5S rRNAs less stable than simulated sequences.

### Compensatory Evolution in 5S rRNA Sequences

Using CoMap methods, we detected 24 and 27 two-site groups of coevolving sites for eubacteria and eukaryota, respectively (Table S2). At least 91.6% of them were located within the known structural regions. Moreover, a total of 23 and 35 of significant coevolving sites pairs were detected by MIp methods for eubacteria and eukaryota, respectively (Table S2), almost 88.6% of which were already known structure pairs. In our analyses, only pairs that were retrieved by both the CoMap and MIp methods were considered as true “coevolving pairs” (Table 1). In eukaryota, most of compensatory changes occurred in the helix I, helix III and helix IV adjoining the loops or bulges (Table 1). The result was slightly different in bacteria as many compensatory changes were found to have occurred in helix I and helix IV, but few in helix III.

We observed the pattern of paired sequence differences through examination of the changes that occurred between nodes (branching points) on the phylogenetic trees. Figure 4 shows an example of coevolving site pairs in animal sequences. The closely related species allowed us to detect most of the intermediate states (e.g. GU). We observed 299 compensatory substitutions of all four types, with 66.6% of them belonging to the AU↔GC type (199 cases, Table 2) and 27.6% belonging to the GC↔CG type. The other two compensatory switches were very scarce with few intermediate states, as shown in Table 2. The prevalence of errors caused by multiple substitutions at each sites in our results must be low, as only 4% of switches involved multiple substitutions.

For each deleterious mutation in a stem, we observed more than two potential patterns of compensation, one involving restoration of the Watson-Crick interaction (available for almost all mutations) and, more than one indirect change (Figure 5). We observed three patterns of compensation altogether. Beside the second-site substitutions that directly restore the disrupted Watson-Crick interaction, we noted several indirect mechanisms of coevolution. First, multiple changes could compensate for one deleterious mutation. As shown in Figure 5, the loss of an A24U pair in helix III was compensated by the mutations A27→G and U52→C that created a GC pair in the same helix. Second, compensation could occur by creating new Watson-Crick pairs. For example, the mutation C69→U in the helix V that disrupted a GC pair was compensated by a C103→A substitution that created an extra AU pair in the same stem, thus reversing the loss of free energy. Another mechanism of compensation may involve the interaction of neighboring pairs within a stem or the interactions of stem and loop structures. For example, the compensatory changes that created an extra Watson-Crick pair in helix V also eliminated loop E. Our results indicated that compensatory evolution might involve complex interactions between multiple sites.

### Positive Selection through the Evolution of 5S rRNA

**Figure 7:**
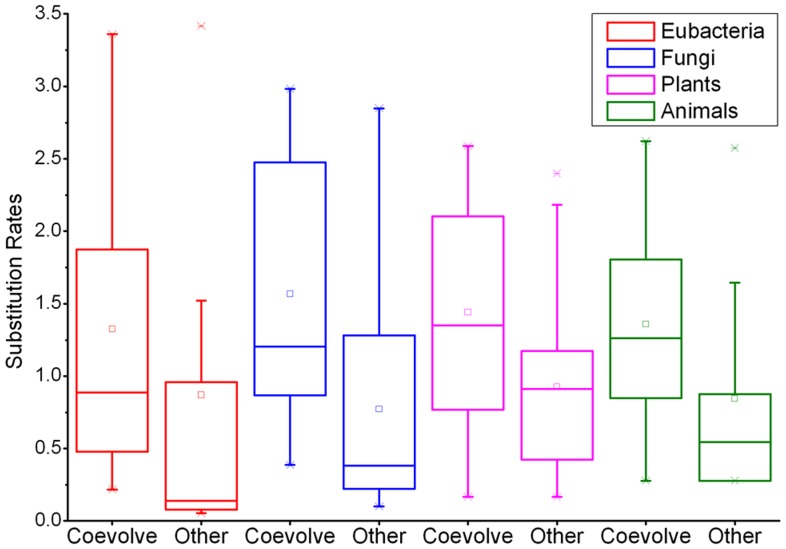
Overall rates of evolution for different stem regions.

As shown in Table 3, analysis of the 5S rRNA sequences demonstrated that there was a significant (p<0.01) excess of base-paired differences in the observed distributions over that expected by chance. In general, the observed value of paired differences was two or three-fold larger than that predicted by the neutral model. As shown in figure 6A, there was a decrease in unpaired bases within stems during the evolution of 5S rRNA, which may lead to an increase in RNA stability. The phylogenetic tracing of structural transformation confirmed that 5S rRNA molecules evolved to attain high conformational order (Figure 6B). This observation was significant different from the data sets simulated by both Seq-Gen and PHASE (Figure 6C, p<0.002 for Seq-Gen and p<0.01 for PHASE). We found that all but one (*S.nova*) simulated sequences were less stable than native 5S rRNAs (Table 4). Taken together, our findings suggest that compensatory evolution results, somewhat indirectly, from natural selection in favor of thermodynamically stable RNA structures. Our structural analysis showed that natural selection occurred early in the evolutionary history of 5S rRNA. Besides, we found a significant difference in substitution rates between the coevolving pairs and other stem pairs, demonstrating distinct evolutionary constraints (Figure 7). We observed 255 cases of compensatory changes in which LCA states were identical to the terminal states and 44 cases in which the LCA states were identical to an intermediate states (Table 2), resulting in a TIR of 5.8∶1. For the sequences simulated by Seq-Gen, we observed a TIR of 1∶1.17 (98,135 terminal LCA state: 114,545 intermediate states). The TIR at interacting sites was about 7 times that of our simulated data, and the difference was statistically significant (p<0.001, Fisher’s t-test). The nonrandom occurrence of paired differences, the high rate of compensatory evolution, and the high TIR scores together indicate that positive selection was involved in the evolution of 5S rRNA sequences.

## Discussion

In this paper, we provide a novel perspective about the effect of selective constraints on the evolution of RNAs. Previous studies have shown that base pairing constraints are the main driving force of evolution in stem regions of RNA molecules [43], [53]. We provide new evidence that sequence sequence coevolutionary interactions are highly dependent on secondary structure, and demonstrate that the compensatory evolution results from natural selection in favor of thermodynamically stable structure.

Compensatory substitutions often occurred within helices that were crucial for the function of RNAs. Helix I and helix IV of 5S rRNA are necessary factors for its mitochondrial targeting. Thus, mutations that maintain base pairing in helix IV will improve 5S rRNA import efficiency, while destabilizing mutations in this region not only affect the structure but also decrease import efficiency [54]. Although disruption of helix I only slightly changes 5S rRNA importability, mutations that interrupt stacking inside the helix will decrease the rate of re-association of ribosomal subunits [54]. Helix II and helix III are important for protein binding and RNA interactions, so destabilizing mutations in these regions strongly affect the translation accuracy and may even be lethal [55], [56].

The occurrence of wobble pairs and mismatch-pairs in the helices indicates that the strength of selection may vary substantially among base pairs. The rate of compensatory changes also depends on the structural features of the molecule, as pairs adjoining loops or bulges may be under different selective constraints compared to internal pairings [57], [58]. The only coevolving positions that were not near a loop or bulge were in helices I and IV in both Eukaryotes and Eubacteria. The likely explanation for this observation is that helices I and IV are longer than other helices, and selective constraints might be relaxed in long helices [59]. Besides, these positions in both helix I and helix IV are adjacent to uncompensated GU pairs, which are known essential for RNA-RNA or RNA-protein interaction [54], [55]. In general, compensatory pairs evolve faster than other stem pairs and may thus be under weaker selective constraints. However, breaking these pairs will directly increase the size of the adjoining loop or bulge, causing a partial or total structural rearrangement. Our results show that most compensatory evolution in 5S rRNAs occur through complex, indirect mechanisms, indicating that previous studies that considered only compensation that restores Watson-Crick pairs were oversimplified.

Our results provide a better understanding of the mechanisms of intra-molecular coevolution in RNAs by incorporating selective constraints of interactions with structural and evolutionary information. Such large-scale analyses should take us towards a general understanding of the coevolutionary processes in RNAs and may even be useful to understand the functional and structural interaction of complex molecules.

## Supporting Information

Figure S1 **The phylogenetic tree of fungi (A) and plant (B).**
(TIF)

Table S1 **Ancestral state reconstructions and their TIR values of compensatory substitutions using different models and methods.**
(DOC)

Table S2 **Coevolving sites detected by CoMap and MIp methods.**
(XLS)
