# Supplementary figures and images for: Coevolution in RNA Molecules Driven by Selective Constraints: Evidence from 5S rRNA
Source: PLoS One. 2012 Sep 4;7(9):e44376. doi: 10.1371/journal.pone.0044376 (PMC3433437; doi:10.1371/journal.pone.0044376)

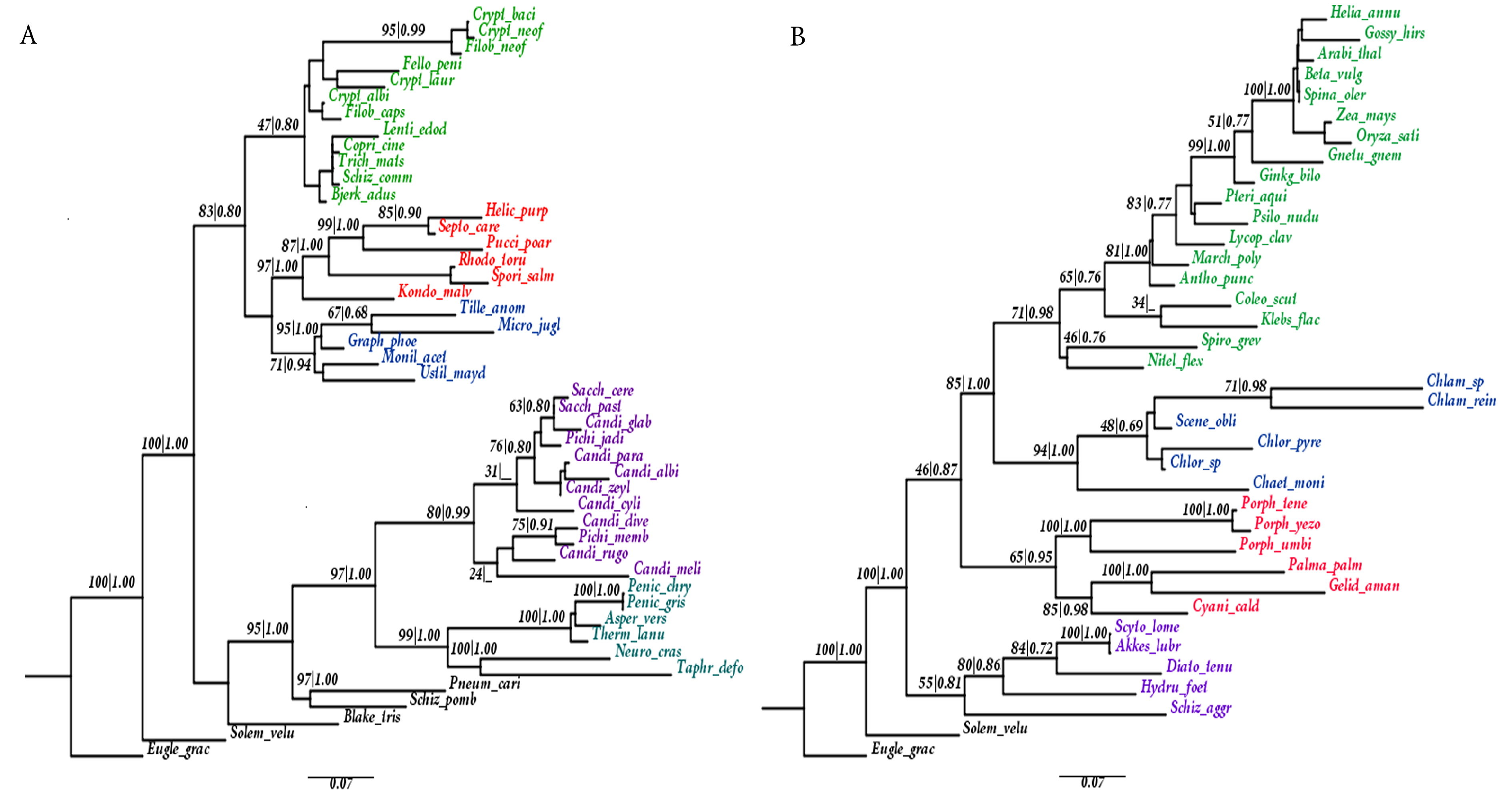

Supplement: Figure S1 — The phylogenetic tree of fungi (A) and plant (B). (TIF) [file pone.0044376.s001.tif]
