# Supplementary material for: Coevolution in RNA Molecules Driven by Selective Constraints: Evidence from 5S rRNA
Source: PLoS One. 2012 Sep 4;7(9):e44376. doi: 10.1371/journal.pone.0044376 (PMC3433437; doi:10.1371/journal.pone.0044376)
Supplement: Table S1 — Ancestral state reconstructions and their TIR values of compensatory substitutions using different models and methods. (DOC) [file pone.0044376.s002.doc]

| Species | Models | Differences | | Ancestral state(Terminal) | Ancestral state(intermediate) | TIR |
| --- | --- | --- | --- | --- | --- | --- |
|  |  | Paired | Unpaired |  |  |  |
| Eubacteria | REV(GTR) | 20 | 5 | 43 | 15 | 2.9:1 |
|  | JC69 | 20 | 4 | 44 | 15 | 2.9:1 |
|  | K80 | 23 | 7 | 43 | 15 | 2.9:1 |
|  | F81 | 20 | 5 | 43 | 15 | 2.9:1 |
|  | F84 | 20 | 5 | 43 | 15 | 2.9:1 |
|  | HKY85 | 20 | 5 | 43 | 15 | 2.9:1 |
|  | T92 | 19 | 5 | 43 | 15 | 2.9:1 |
|  | TN93 | 20 | 4 | 43 | 15 | 2.9:1 |
| Fungi | REV(GTR) | 38 | 15 | 103 | 15 | 6.9:1 |
|  | JC69 | 38 | 15 | 102 | 17 | 6.0:1 |
|  | K80 | 38 | 15 | 101 | 19 | 5.3:1 |
|  | F81 | 38 | 15 | 101 | 19 | 5.3:1 |
|  | F84 | 38 | 15 | 101 | 19 | 5.3:1 |
|  | HKY85 | 38 | 15 | 103 | 15 | 6.9:1 |
|  | T92 | 38 | 15 | 101 | 19 | 5.3:1 |
|  | TN93 | 38 | 15 | 101 | 19 | 5.3:1 |
| Plants | REV(GTR) | 15 | 1 | 54 | 6 | 9.0:1 |
|  | JC69 | 13 | 2 | 53 | 6 | 8.8:1 |
|  | K80 | 14 | 0 | 54 | 6 | 9.0:1 |
|  | F81 | 15 | 1 | 54 | 6 | 9.0:1 |
|  | F84 | 15 | 1 | 54 | 6 | 9.0:1 |
|  | HKY85 | 15 | 2 | 54 | 6 | 9.0 :1 |
|  | T92 | 16 | 3 | 54 | 6 | 9.0:1 |
|  | TN93 | 13 | 4 | 54 | 6 | 9.0:1 |
| Animals | REV(GTR) | 19 | 8 | 55 | 8 | 6.9:1 |
|  | JC69 | 19 | 8 | 55 | 8 | 6.9:1 |
|  | K80 | 19 | 8 | 55 | 8 | 6.9:1 |
|  | F81 | 19 | 8 | 55 | 8 | 6.9:1 |
|  | F84 | 19 | 8 | 55 | 8 | 6.9:1 |
|  | HKY85 | 19 | 8 | 55 | 8 | 6.9:1 |
|  | T92 | 19 | 7 | 55 | 8 | 6.9:1 |
|  | TN93 | 19 | 8 | 55 | 8 | 6.9:1 |
